# Public challenge and endorsement of sex category ambiguity in online debate: ‘The sooner people stop thinking that gender is a matter of choice the better’

**DOI:** 10.1111/1467-9566.12490

**Published:** 2016-11-16

**Authors:** Helen Sweeting, Matthew William Maycock, Laura Walker, Kate Hunt

**Affiliations:** ^1^ MRC/CSO Social and Public Health Sciences UnitUniversity of GlasgowScotland

**Keywords:** sex, gender, lay concepts, Internet and research, content analysis

## Abstract

Despite academic feminist debate over several decades, the binary nature of sex as a (perhaps *the*) primary social classification is often taken for granted, as is the assumption that individuals can be unproblematically assigned a biological sex at birth. This article presents analysis of online debate on the BBC news website in November 2013, comprising 864 readers' responses to an article entitled ‘Germany allows ‘indeterminate’ gender at birth’. It explores how discourse reflecting Western essentialist beliefs about people having one sex *or* ‘the other’ is maintained in debates conducted in this online public space. Comments were coded thematically and are presented under five sub‐headings: overall evaluation of the German law; discussing and disputing statistics and ‘facts’; binary categorisations; religion and politics; and ‘conversations’ and threads. Although for many the mapping of binary sex onto gender was unquestionable, this view was strongly disputed by commentators who questioned the meanings of ‘natural’ and ‘normal’, raised the possibility of removing societal binary male‐female distinctions or saw maleness–femaleness as a continuum. While recognising that online commentators are anonymous and can control their self‐presentation, this animated discussion suggests that social classifications as male or female, even if questioned, remain fundamental in public debate in the early 21^st^ century.

## Introduction

In November 2013 the BBC news website reported the passage of a new law in Germany that overtly challenged the dichotomous classification of newborns as either ‘male’ or ‘female’. The news article, (BBC News, 2013), described Germany as ‘Europe's first country to allow babies with characteristics of both sexes to be registered as neither male nor female’, so creating ‘a new category of “indeterminate sex”’. The article precipitated 864 reader comments posted on the website. We argue that these provide a rare chance to examine public understandings and views of ‘sex’ and ‘gender’ and the extent to which they reflect longstanding academic debates about these phenomena as unambiguously dichotomous (or not), as overlapping or distinct, and as fluid or fixed. The comments include the perspectives of people with relevant life experiences or self‐proclaimed vested interests and others who may have no specialist knowledge of the issues involved. To contextualise this analysis, we first briefly rehearse relevant and well‐recognised problems with the terms ‘sex’ and ‘gender’ and provide perhaps less familiar background on how the naming of what the BBC referred to as ‘indeterminate sex’ is also ‘politicized and controversial’ (Davis 2015: 89) and historically bounded.

### Sex and gender: distinguishable and distinct?

Since the 1970s, following Oakley's popularisation of a distinction between sex (‘the biological differences between male and female: the visible difference in genitalia’) and gender (as cultural, ‘the social classification into ‘masculine’ and ‘feminine’) (Oakley 1985:16), the extent to which these can be distinguished conceptually and empirically and the pervasiveness of binary thinking about both sex and gender have been challenged by many authors, including Oakley herself (1985). In this long, contested, academic debate (for example, Butler 1990, Fausto‐Sterling 1993), contrasts have been made between ‘having’ a sex and ‘being’ a gender (Hester 2004b, Paechter 2003), between sex as biology and gender as social or socially constructed (Rubin 2012). Lorber and Farrell highlight societal investment in gender categorisation, noting how gender ‘is built into the social order … The major social institutions of control – law, medicine, religion, politics – treat men and women differently’ (1991: 1–2). Their description of gender as ‘a major social status (if not *the* major social status)’ (p. 2) echoes Goffman (1977: 302), who asserted that ‘In all societies, all infants at birth are placed in one or in the other of two *sex classes,* … accomplished by inspection of the infant's naked person, specifically its genitalia, these being visibly dimorphic’ (emphasis in original), arguing that what he termed ‘sex‐class placement’ is ‘*almost without exception exhaustive of the population and life‐long*, providing an exemplary instance, if not a prototype, of social classification’ (emphasis added, p. 302).

Goffman's description of the ubiquity of ‘sex‐class placement’ at birth provides an obvious context for the online discussion provoked by the new German law. However, not only has the uncritical (or interchangeable) use of terms in academic publications and analyses blurred the distinction between sex and gender (Emslie *et al*. 1999) but it is increasingly recognised that they do not map neatly onto each other (Krieger 2003) and that, despite assertions that ‘It is very easy to classify people according to their [biological] sex’ (Alvesson and Due Billing 1997: 26) this is by no means universally true.^1^

### The changed and changing language and categorisation of ‘intersex’/DSD

The BBC article uses the term ‘intersex’ to refer to babies born with what is currently, in medical contexts at least, termed ‘disorders of sexual development’ (DSD), following a consensus conference held in Chicago in 2005 that defined DSD as ‘congenital conditions in which development of chromosomal, gonadal, or anatomic sex is atypical’ (Lee *et al*. 2006: e488). It is often described as an umbrella term, covering a wide range of conditions defined by the social meaning attached to atypical sex anatomy rather than common causes or clinical features (Karkazis and Feder 2008).

The language used to describe those with such conditions has been contested over many decades (Davis 2015). The term intersex was introduced in the early 20^th^ century to refer to ‘biological sex types that fell between male and female’ (Dreger and Herndon 2009: 208) and to ‘describe the state of being born with a combination of characteristics (for example, genital, gonadal, and/or chromosomal that are typically presumed to be exclusively male or female’ (Davis 2015: 2). The term became associated with an all‐encompassing identity (as neither or both male and/or female) and with political activism, and some argued that it was disliked by many of those personally affected (Dreger and Herndon 2009, Feder and Karkazis 2008). Those who support the use of the term DSD believe it emphasises the biological factors impacting on sex development, rather than identity, and so may be more manageable and less stigmatising than alternatives (Karkazis and Feder 2008); indeed, it has been suggested that most parents (95%) and healthcare professionals (80%) prefer the term (Davies *et al*. 2011). However, others see DSD (particularly ‘disorder’) as medicalising or pathologising (Davies *et al*. 2011, Reis 2007).

While DSD is currently used in much of the medical literature to which we refer, we recognise arguments by Davis (2015) that its use has caused tension in the intersex community, and those rejecting ‘DSD language tend to reject the idea that sex, gender and sexuality are biologically prescribed bodily phenomena’ (p. 146). We have tried to respect views that ‘people should be able to choose whatever term – or terms they find suitable’ (Davis 2015: 146) by using ‘intersex/DSD’ in what follows (except in direct quotes from articles or online comments). In doing this we seek to signal our recognition that the ways such terms are (re)appropriated by different protagonists are very specifically socially and historically located.^2^ We also note that some who reject the pathologisation of people labelled as intersex/DSD may prefer to read DSD as ‘divergences’ of, rather than ‘disorders’ of sex development (Feder and Karkazis 2008, Reis 2007).

### Rates and medical management of intersex/DSD

Estimates of intersex/DSD rates differ widely, due to both the secrecy or stigma that are often associated with any uncertainty about ‘sex‐placement’, and the between‐population variations in the rates of some intersex/DSD conditions (Ahmed *et al*. 2004, Blackless *et al*. 2000, Chau and Herring 2002, Dreger and Herndon 2009). However, the main reason for differing estimates is variation in what ‘counts’ as intersex/DSD. In 1993, Fausto‐Sterling (1993) reported that the psychologist Money, who specialised in the study of those born with sexual‐organ ‘defects’, had suggested that ‘intersexuals’ may constitute up to 4% of births. The immediate refutation and description of the statement as ‘epidemiologically reckless’ by Money himself (Money 1993) appears to have been overlooked, and the 4% figure has been repeated in the literature (Chau and Herring 2002, Gough *et al*. 2008, Zeiler and Wickstrom 2009). A review of over 40 years’ medical literature, conducted in 2000, concluded that 1.7% of all live births did not conform to absolute sex chromosome, gonadal, genital and hormonal dimorphism (Blackless *et al*. 2000). However, this very broad definition includes individuals whose genitalia appear ‘normal’ at birth, and a subsequent article suggested that restricting it to those who would be recognised by clinicians as having intersex/DSD at birth, reduces the prevalence to around 0.018% (Sax 2002). Applying these figures to the UK's 777,400 births in 2014 (Office for National Statistics 2015) would result in estimated numbers of babies born with intersex/DSD that year of 31,100 (4% births), 13,200 (1.7%) or 140 (0.018%).

Before the 20^th^ century there was no medical management of intersex/DSD (Preves 2002). In 1955, Money and colleagues proposed guidelines that for the next 40–50 years dominated the medical approach to children born with what has often been described by both clinicians and activists (for example, Blizzard 2002, Dreger and Herndon 2009, Hughes 2008) as ‘ambiguous genitalia’ (Chau and Herring 2002, Hester 2004a). These guidelines were premised on the belief that we are born ‘psychosexually neutral’ and that ‘children could be steered one way or the other so long as the steering began before the age of two, give or take a few months’ (Dreger and Herndon 2009: 202). Over this period a newborn with ‘ambiguous genitalia’ was typically treated as a medical ‘emergency’, to be considered by a clinical team (physician, endocrinologist, urologist and possibly also psychologist/psychiatrist) who tried to determine the child's ‘true sex’ based on examinations and tests to determine presumed future fertility, endocrine function and pubertal development (Hester 2004b, Kuhnle and Krahl 2002). Surgical intervention, usually directed to constructing genitalia as female (Barbaro *et al*. 2011, Kessler 1990), was recommended as soon as possible, to create genitalia compatible with the ‘sex of rearing’ (Ahmed *et al*. 2004, Barthold 2011, Blizzard 2002). Before deciding whether to consent to such surgery on behalf of their child, parents were generally advised not to use gender pronouns when referring to them. After deciding, typically the child was given a gendered name, received surgery, and parents were asked to consistently socialise them in line with their surgically modified anatomy (Hester 2004a).

By the 1990s Money's hypothesis and the resulting paternalistic medical approach, particularly early surgery, was increasingly challenged (Chau and Herring, 2002). Methodological limitations meant the evidence justifying its continuation was weak (Barthold 2011) and one key piece of evidence, the so‐called ‘John/Joan’ case, was shown to be flawed. In this infamous case, one of a set of twin boys suffered severe burning to his penis during surgical treatment. His parents sought Money's advice, who recommended the child should have the remainder of his penis removed, this operation being carried out at around the age of 20 months (Money and Ehrhardt 1972). Following his reassignment as a girl, Money instructed the family to name, and treat, him as female. Although this ‘experiment’ was initially presented as successful and evidence of Money's theory (Chau and Herring 2002, Dreger and Herndon 2009, Preves 2002), ‘Joan’ subsequently reported the immense difficulties he experienced until he eventually rejected his assigned female name (to become David Reimer) and body (receiving surgery to reconstruct a penis) (Colapinto 2000). Around the same time there was growing activism of adults who had received surgical treatment as infants, raising doubts about the consequences of unnecessary (or unnecessarily early) interventions performed without the patient's informed consent (Hegarty and Chase 2000). Partly in response to this, healthcare professionals also began questioning the need for early surgery and focused increasingly on patient‐centred care (Barthold 2011, Reis 2007); and a ‘physician‐patient covenant’ (Rivkees 2006: 1287). The 2005 Chicago Consensus statement therefore noted that appearance‐altering surgery was not urgent while also recommending rapid gender assignment based on open communication between a multidisciplinary team and the baby's family (Houk *et al*. 2006). A 2016 update on the diagnosis and care of individuals with intersex/DSD notes the continued controversy around medical management and ‘intense scrutiny’ of surgical intervention (Lee *et al*. 2016), with some studies suggesting there was only very slight evidence for practice changes in childhood surgery for ambiguous genitalia since the publication of the consensus (Michala *et al*. 2014).

### Problematising binary sex/gender categorisations

However, more fundamental doubts of some scholars over the medical management and ‘normalisation’ of those born with ‘ambiguous genitalia’ were not addressed by the Chicago Consensus statement. These can be summarised as the perpetuation by the medical community of ‘the belief that gender consists of two exclusive types… in the face of incontrovertible physical evidence that this is not mandated by biology’ (Kessler 1990: 25). Paralleling the more general medicalisation literature that highlights how healthcare systems reflect societal values in their categorisation and correction of ‘abnormal’ bodies (Brown 1995), these authors point out that binary gender norms are so universal in Western cultures that they are internalised as ‘natural’, with the resulting pathologisation of bodies deviating from the norm (Bishop 2007, Dreger and Herndon 2009, Fausto‐Sterling 2000, Feder and Karkazis 2008, Hester 2004a). Those critical of past medical management strategies suggest they focused on fixing intersex/DSD, when it is the social system which is reductive and pathological (Preves 2002).

Just as defining children as abnormal in relation to height and weight growth charts has been problematised (Armstrong 1995), so authors questioning binary gender norms have suggested that babies born with intersex/DSD demonstrate that the area between complete ‘maleness’ and complete ‘femaleness’ is natural (Chau and Herring 2002, Fausto‐Sterling 2000). As discussed above, sex‐class placement and its presumed overlap with gender (and often also sexuality [Davis 2015]) is the foundation of Western social structures, a basis of self‐identification and societal organisation (Goffman 1977, West and Zimmerman 1987). However, this need not be the case; there are few situations where the law needs to distinguish male from female (Chau and Herring 2002), and in different historical periods and cultures more than two sex categories have been recognised (Lang and Kuhnle 2008, Monro 2007). Some suggest an alternative might be to dissolve the distinction between male and female (Fausto‐Sterling 2000), conceptualising sex as a continuum (Monro 2007).

However, despite a socio‐political context that is much more open to a range of sexual identities than previously (Roen 2004), even those considering a future without distinction on the grounds of sex find it difficult to imagine in current circumstances (Warnke 2001). In cultures where more than two sex categories are recognised, the status of the minority categories tends to be low (Ahmed *et al*. 2004). Crucially, some individuals affected by intersex/DSD do not believe that shame or stigma will necessarily be reduced by raising children as a third or no gender (Dreger and Herndon 2009) and the few studies of parents of babies born with ‘ambiguous genitalia’ highlight their bewilderment and disorientation, which is relieved only when their baby is assigned a sex (Gough *et al*. 2008, Zeiler and Wickstrom 2009).

### This study

It is against this contested debate both about sex/gender and the diagnosis, naming (Davis 2015) and societal ‘conspiracy of silence’ (Kerry 2011) about intersex/DSD that we set our analysis of readers’ comments responding to the BBC's story about the introduction in Germany of a category for ‘indeterminate sex’ at birth. It has been suggested that while the media can provide information and shape responses to issues (Kitzinger 2000, Seale 2002), ‘the term intersex fails to make its mark in the media’ (Kerry 2011: 263). Two exceptions to this general rule prior to the BBC piece followed revelations of David Reimer's rejection of his imposed female gender (Colapinto 2000) and the reporting in 2004 of his suicide.^3^ Another crucial aspect of context is the growth of the Internet since the mid‐1990s, enabling (private) online information searches (Dutton *et al*. 2013) and user‐generated content, including blogs and commentaries (Hookway 2008, Jönsson and Örnebring 2011). It has been suggested this has been the key to the emergence of the intersex movement (Kerry 2011) and to diminishing the social isolation of intersex/DSD people and their parents (Davis 2015). Our analysis allows us to explore how discourse reflecting essentialist beliefs about people having one sex *or* ‘the other’ is maintained in debates in this online public space (Bou‐Franch 2013).

## Methods

The BBC article noted that parents of babies born in Germany were to be allowed to leave gender blank on birth certificates, ‘in effect creating a new category of “indeterminate sex”’. It suggested that as ‘many as one in 2,000 people have characteristics of both sexes’ (implying a 0.05% prevalence rate) and described ‘intersex’ people as having a mix of male and female chromosomes or genitalia characteristic of both sexes. The article highlighted parental difficulties in having to quickly choose which sex to register their baby,^4^ described the harmful long‐term effects of surgery performed on babies and quoted an ‘intersex’ woman and counsellor as saying ‘This pink and blue thing is a nonsense’. It also noted that several countries had taken similar steps, beginning with Nepal, which recognised a third gender on census forms in 2007 and including Australia, New Zealand and Bangladesh (passport applications), Pakistan (national identity cards), India (voter lists) and Thailand (official recognition by the military).

**Figure 1:**
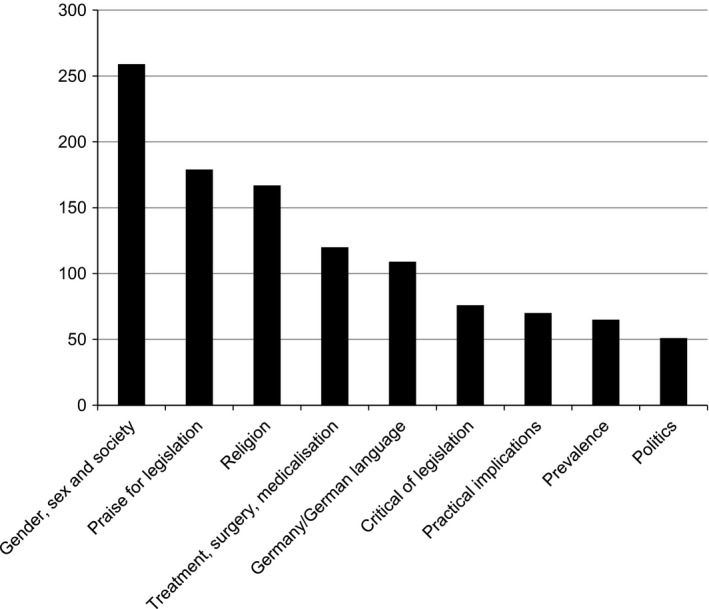
Number of comments coded to each theme

Altogether 864 comments were posted within 12 hours of the appearance of the article on 1 November (08:29 to 19:59, when the entry was closed to comments). Of these, 36 were removed by the website moderator for contravening house rules.^5^ The remaining 828 comments were coded thematically, based on their interpreted meanings (rather than the use of explicit words or concepts), using NVivo 9. Following discussions among all authors of the initial themes, three (LW, MM, HS) independently coded the first 100 comments and agreed on the following: specific praise/criticism of legislation; prevalence of intersex/DSD; gender, sex and society (including ‘facts’/understandings and opinions/debate); practical implications; treatment, surgery and medicalisation; religion; Germany and politics. Some comments were coded to several themes; comments responding to other commentators were also identified. A further 100 comments were independently coded by two researchers (LW, MM), as an iterative process until complete agreement was reached; LW then coded the remainder. The relatively small amount of textual material (around 40,000 words) meant all coding could also be verified by HS during analysis and writing up the results. Figure 1 shows the number of comments coded to each theme.

The default format for BBC comments is the comment number, commentator name and their comment. Responses to specific comments are prefaced by the commentator number and name to whom they are being addressed. This means conversational threads are identifiable, even when separated by unrelated comments, unless an individual chooses to remove the name of the commentator to whom they are replying. The most prolific commentators and their ‘conversations’ were identified by searching for their names as a commentator or within others’ replies.

Following a brief descriptive introduction, we present our analysis under five sub‐headings: overall evaluation of the German law; discussing and disputing statistics and ‘facts’; binary categorisations; religion and politics; and ‘conversations’ and threads. Examples of comments are presented as originally posted, including any grammatical or spelling errors, and are unattributed.

## Results

The 864 comments were uploaded by 493 individual commentators, with 366 making only one comment. The 10 most prolific together contributed 151 comments. Although the potential for anonymity and control over self‐presentation means we cannot know anything for certain about those posting (including their gender), some comments were very personal:

I'm a man, married with kids (donor) and (drum roll) intersex… I have an extra X chromosome. The biggest barrier faced by intersex men, women and children are some of the views below [referring to comments already posted]; it was not until I accepted that I am mixed gender that I actually found happiness.

Other commentators referred to the experiences of people they knew, including a child, sibling (‘My brother was born in the early 60s of indeterminate sex and Drs chose that he would be male less than 2 hours later after a ‘“thorough look‐see”’), friend, colleague and ‘a family’ in what may have been a professional encounter (‘I had a distressing meeting with a family in this circumstance a few days ago’). Sometimes more general references to ‘people I know of’ were wielded to counter arguments that intersex/DSD is extremely rare.

### Overall evaluation of the German law

Around a fifth of comments were specific in their praise of the law (‘good’, ‘well done’, ‘not a day too soon’, ‘common sense’), outnumbering specifically critical ones (‘rubbish’, ‘madness’, ‘a laugh’) by over two to one. Comments in praise of the law could be divided into two types. The first included those describing it as a ‘sensible’ solution to a practical problem: ‘To all of you judging this a stupid: Imagine how it would be for you if it were your own child. Done? Thought so’. The rationale was that parents and doctors should not be forced to make rushed decisions; that surgery based on ‘guesswork’ about the most ‘appropriate’ sexual organs should not be imposed on those unable to consent; and that individuals should be able to choose their sex/gender (both terms were used) when they were old enough: ‘Hippocratic oath says first do no harm, aka, engage brain before cutting newborn for the sake of burocracy’. Most such commentators believed the child would behave or identify as male or female with time: ‘as they get older they will veer towards one sex or the other’. Only a very small number suggested that ‘a dominant gender [might not] prevail’. The second main type of positive comment praised the law as ‘progressive’, tending to focus on broader societal issues ‘[it] is a small step towards a more general acceptance that the gender is not strictly defined by the body it's in’; ‘Next, no gender labels except the ones we chose as individuals’.

Among the critical comments were some expressing incomprehension about the need for a ruling: ‘What's the point in this? The only genders are male or female’; ‘The common sense view of course is… Two bloody genders’; ‘Ridiculous – they either have a Y chromosome or not – end of story. The sooner people stop thinking that gender is a matter of choice the better’. A small number thought the law was unnecessary as it would apply to so few people. A larger group of critics regarded it as evidence of an overly liberal or ‘sick’ society: ‘An entirely new category they can investigate to check for discrimination, demand new resources, protect their jobs’. A few, however, criticised the new law for not going far enough, ‘There needs to be a ban with possible prison sentencing for any one that takes part on bringing a scalp[el] near a child's genitals’. Other critical comments came from those who felt the new law would not solve practical problems, ‘Words are just words. They will still have to choose which toilet to go in’; ‘adding a new ‘gender’ don't solve the problems, it add another complexities’.

### Discussing and disputing statistics and ‘facts’

The statement in the BBC article that ‘As many as one in 2,000 people have characteristics of both sexes’ prompted discussion. Some accepted the figure, generally expressing surprise (‘I had no idea it was so common’) or relating it to other disorders (‘the % of people born with this… is higher than that of those born blind’). Although these commentators generally suggested the German law was justified, some thought ‘a whole new category’ for such a small percentage of the population was unreasonable. Only two comments questioned the figure as too low, referring to Intersex Society of North America website figures of 1 in 100 newborns with bodies differing ‘from standard male and female’. Far more suggested the figure was too high. Some simply could not believe it, apparently assuming that intersex/DSD would be obvious to all in the social networks of those affected (‘I find that hard to believe or we would all know someone like this’) or noted that the BBC wording of ‘as many as…’ implied the number born with intersex/DSD was probably (much) smaller. Some responses to these ‘too high’ comments quoted statistics on various disorders. Others noted that not all those with intersex/DSD required surgery or were easily identifiable: ‘you pass these people every day in the street, but they don't advertise it’. An alternative set of responses suggested issues of prevalence were less relevant than issues of sensitive treatment: ‘Even if it only affected 1 in 50,000 babies this [the German law] would be the sensible thing to do’.

Almost one in ten comments included debate about ‘facts’ relating to chromosomes and the biology of sex and intersex/DSD. Many were responses to essentialist suggestions early in the thread that people are either ‘male or female. Fact’ or that ‘one DNA test can reveal whether you are male or female within the hour’. A few expressed surprise that this was not the case: ‘I thought there were only boys and girls’; others wondered how a third gender would be distinguished at birth. Some of these comments included the general idea that people are ‘born hermaphrodites’, thus ‘medically both genders’ and that ‘biological sex is just not as cut and dried as people think’. Others included more specific details, referencing, recommending or including links to various websites.

Most commonly, such comments noted chromosomal combinations other than XX or XY: ‘one X only (turner syndrome – under‐developed female), XXY (Klinefelters – looks male with female characteristics)’. Similar comments suggested ‘chromosomes are only part of the equation’, that hormonal influences (‘the androgen bath’) also determine whether a child is born male or female, that genes may be switched on or off, chromosomes may not ‘behave as normal’ or that ‘you can have some cells with a Y and some without in the same body’. There was mention of early foetal sexual differentiation, anatomical similarities between male and female genitalia and comments that while someone might appear ‘normal’ they could have atypical internal sexual organs: ‘if you happen to be born with a penis but also ovaries and a vagina’.

### Binary categorisations

There was more general debate around the binary male‐female categorisation, encapsulated in one comment, ‘as it was, and always will be boys and girls’. Some suggested that those with intersex/DSD do not constitute a third gender since this would require ‘sexual attributes that are neither male nor female’, but rather both (‘checkmark both boxes’) or neither (‘is it… ‘none of the above’?’). Many drew distinctions between sex and gender (‘Your SEX is defined by your chromosomes… Your GENDER is what you want it to be’ [capitals in original]; ‘Sex is the correct term. penis = male vagina = female something that looks odd = Intersex… but gender is established in the brain’). A smaller number discussed social roles (‘social gender’; ‘a social construct’). Some also suggested that there are already more than two genders while others proposed that gender is, or should be, ‘a sliding scale’. For a small group, such suggestions were laughable. These commentators derided the BBC (‘people are formed in many genders… this is our faith and we ask the bbc to respect it’), recalled old ‘jokes’ (‘They're going to have three children, one of each’), or made up new ‘jokes’ of their own (‘‘Bizarre’ might be a good choice for a name in the circumstances’).

Almost one in ten comments related to the practical implications, most frequently choice of toilet, changing rooms, names and how those with intersex/DSD might be referred to, clothing colour or type, room décor. They also referred to the possibility of bullying, the implications of ‘X’ on a passport and how such individuals would be categorised for sports participation. Most of these were framed as questions (flippant or serious), for example, ‘[toilet] seat up or seat down?’, ‘how would we refer to a third gender respectfully since ‘it’ is used for inanimate objects, but we are referring to a person?’ Some of these questions generated practical responses including all‐cubicle unisex toilets, and gender‐neutral names. Others used their response to make more general points relating to the need for binary distinctions, ‘is there ANY circumstance in the modern world where biological gender really matters?’, or reasons for their perpetuation, ‘children are very able to understand until the adults have taught them to hate anyone different’. A small number were stronger: ‘Maybe they need to create a new toilet to keep bigots and racist people separate’.

Around one in ten comments also questioned the necessity of sex or gender‐based categorisations, which they perceived as constructing those with intersex/DSD as a societal problem. This group did not share the view that ‘a third gender is creepy and unnatural’, but located ‘the problem’ as societal: ‘the problem is not them but our acceptance of what they are’. Within these comments was discussion of what was ‘natural’ and ‘normal’, with commentators suggesting that biology or ‘nature… produces people like this’ so, although they are unusual, they could not be ‘unnatural’. In response to those suggesting that although natural, it was a ‘mistake’ and thus a ‘deformity’ or disability, since such people ‘cannot spawn young’, a very small number invoked the idea of forms different from those expected by society. More broadly, this group viewed ‘unnecessary’ ‘genital mutilation’ of newborns to conform to ‘ignorant and prejudiced’ societal expectations as unethical. It thus followed that postponing categorisations until the child was old enough for ‘individual choice’ was morally and practically preferable. Most who referred to those with intersex/DSD in this context appeared to assume the eventual choice would be between male or female, thus ‘keep[ing] the categories as per the original divine blueprint’. However, many discussed the issues more generally, advocating removal of ‘male’ and ‘female’ from official documents and suggesting that, apart from certain medical issues, there is no legal or official ‘need [to] know what is between any individual's legs’. Like racial, religious or other labels, ‘ludicrous classifications’ based on sex/gender were portrayed as reinforcing discrimination. These commentators argued that in an egalitarian society, we are all simply people: ‘HB denoting human being could be the answer on all birth certificates’. Some suggested that increased publicity and discussion of such issues would reduce prejudice on the basis of sex/gender and sexuality distinctions and that questioning binary categorisations was therefore ‘the human race edging forward sociologically’.

### Religion and politics

Around one in five comments made reference to religion; one‐third of these made no mention of anything related to the BBC story, most were very general and the only religion explicitly mentioned was Christianity. The most directly relevant were in response to two early comments: ‘God created WOMEN from the rib of MAN. God did NOT create another gender. THIS IS BLASPHEMY’ and ‘This will just confuse the children even more. Look to the Lord God our Savior and let Him decide. Man should not be making these sorts of decisions’. Although some suggested these commentators might be trolls,^6^ most suggested children with intersex/DSD should be treated with compassion, that the Bible is not a biology textbook and that the German ruling aimed to prevent adults from making premature decisions (‘“playing God” and imposing a sex on them at birth’), allowing a gender to emerge. Many such responses also made more general comments about religion or expressed distress at extreme religious comments.

A number of commentators made specific reference to the fact that this law had been passed in Germany. A small minority were pejorative, invoking Germany's history, for example, suggesting that perhaps children were born with intersex/DSD in Germany because of previous ‘dabbling with the Aryan dream’. However, the vast majority portrayed Germany as progressive, brave, humanitarian, and a country that could be trusted ‘to do this logically’, in contrast to their ‘dinosaur’ UK or US political counterparts. Several suggested that because the German language includes masculine, feminine and neuter nouns, with babies and children usually referred to as neuter, it was somehow ‘already set up to deal with this’.

Somewhat related politically themed comments condemned the law as a liberal waste of resources, ‘left wing appeasement politically correct nonsense’ and a few extended this criticism to the BBC, ‘the BBC diversity bell must be ring ring ringing’. However, again such comments were countered by suggestions that a ‘sensible’ or ‘humane’ idea was not necessarily left‐wing, minority issues may nevertheless be very important and intersex/DSD should not be politicised.

### ‘Conversations’ and threads

Two of the five most prolific commentators (Alpharius and Inglewood Jack) were critical of the German law. Alpharius (18 comments) entered 18 separate ‘conversations’, five responding to others, arguing against ‘modern feminism’, suggesting ‘intersex children are malformed. They should be helped [and] allowed to decide their true gender, but they aren't a magical third gender that should be celebrated’ and ‘The correct and true should be LGBTQIDZTRSFEDSCJGSLSCUFK8GJF31000101010 so that all minority groups are represented from lesban to robosexuals’. Most of Alpharius’ unprompted comments were removed by the moderator. InglewoodJack (17 comments) engaged in 14 ‘conversations’ in which he argued against ‘trying to be special’ and described himself as ‘100% male … By birth By choice By action By stereotype By every metric I have’. His unprompted comments referred, apparently facetiously, to those ‘trapped in the wrong gender body’ and questioned comments favouring the German law.

Mayna, the most prolific commentator, was responsible for 26 comments in 18 separate ‘conversations’, 14 of which were replies arguing for acceptance of diversity and making strong anti‐religious points. Mayna's unprompted comments raised issues relating to embryonic sexual differentiation and respectful ways to refer to a third gender and asked why so many were concerned about the German law: ‘with the exception of the person themselves, does this actually change anything for the general public – no’. The 13 separate ‘conversations’ involving Bill Walker (14 comments), seven responding to others and six unprompted, were very varied, but largely positive about the German law. He described a colleague who had gender reassignment surgery, non‐gender‐specific names, asked about toilet choice, discussed evolution (‘sorry Bible bashers’), embryo development and the ability of other species to change sex, and suggested Alpharius was ‘a male with a severe attitude problem’. Peter_Sym (13 comments) was involved in 10 conversations, three involving several interactions. Only his first comment was unprompted, responding to the thread so far: ‘Even by HYS [have your say] standards the medically ignorant and god deluded are out in force’. In responding to others he established his credentials as a ‘scientific’ commentator, posited that some corrective surgery may be preferable at in children very young ages, suggested children who are neither male nor female would find school difficult, provided information on chromosomal abnormalities and cloning (‘I do have a degree in genetics and a masters in clinical biochemistry’) and joined in a satirical religious discussion (‘My favourite sport is baiting literal bible fans’).

Almost one in ten comments made general reference to those of others, over a quarter suggesting astonishment or sadness at the tone of comments or low levels of knowledge displayed. A few specifically commented on such comments, ‘This HYS is like a circus. Let's help you ALL out a bit. To the left… 1 in 2000 have these kind of issues at birth?? Rubbish, Bull, Lies. To the right… You can do DNA tests all day long, and no matter what the results the person STILL has both characteristics, your test changes nothing. And to the religious… keep on commenting, absolutely hilarious’, while others questioned the need to respond at all, ‘why do people who are not affected by this get so bothered about it?’

## Discussion

Comments on the ‘Germany allows ‘indeterminate’ gender at birth’ article focus on an issue which has occupied much academic debate: ‘a simple question: why must we have a sex?’ (Hester 2004b: 223). The fact that over 800 comments were posted in under 12 hours, and the disputatious nature of those comments suggests that while the question might appear simple, the answers are controversial (Mishne and Glance 2006), provoking vested interests, discomfort and even anger raised by challenges to the often tacit taken‐for‐granted assumption that humans conform to a binary sex categorisation. Our intention in analysing these comments was to explore how discourse reflecting Western essentialist beliefs about the binary nature of sex is maintained in debates conducted in online public spaces. The points made by many commentators paralleled those in the academic literature to a striking degree. While binary beliefs were voiced, often very strongly, it was something of a surprise that these were outweighed, and strongly disputed, by those with opposing views.

Those expressing an opinion in relation to surgery were almost unanimously consistent with prevailing views (Davis 2015) and the Chicago Consensus (Houk *et al*. 2006, Lee *et al*. 2006) in suggesting delaying ‘unnecessary genital surgery to an age of patient informed consent’ (Houk *et al*. 2006: 755). As several authors point out, most adults with intersex/DSD believe raising a child as a third (or no) gender is socially challenging and suggest it is not gender assignment per se, but associated medical interventions, shame and secrecy which are the problem (Bishop 2007, Davis 2015, Dreger and Herndon 2009, Hegarty and Chase, 2000, Rubin 2012). This was recognised by the many commentators who raised both practical and stigma‐related issues relating to the legislation. Many expressed the view that at some point, maleness or femaleness would emerge via behaviour or individual self‐identification, ‘reflecting common perceptions of sex/gender as an immutable binary biological reality’ (Liao *et al*. 2012: 597).

Commentators debated the meaning of ‘natural’ and ‘normal’ (something can occur rarely but still be natural) in the same way that Sax (2002) takes Fausto‐Sterling (2000) to task for using the word ‘natural’ synonymously with ‘normal’. Again, paralleling academic literature (Chau and Herring 2002, Gough *et al*. 2008, Kessler 1990, Monro 2007, Preves 2002), many online commentators took the discussion a stage further, raising the possibility of removing societal binary male‐female distinctions or seeing maleness–femaleness as a continuum.

The extreme discomfort with such ideas shown by some highlights entrenched inclinations to categorise people as either male or female, and the self‐reinforcing way in which the binary system has become viewed as natural (Dreger and Herndon 2009, Hester 2004b), so natural that it is taken for granted to the point of invisibility. For these commentators, sex *is* scientifically discoverable, akin to the mistaken scientific belief during the 1950s of the ‘Barr body’ as indicating the ‘presence or absence of a female sex chromosome constitution’ (Miller 2006: 260). For this group, those with intersex/DSD are ‘a symbol of boundary blurring: of the anomalous, the unclean, the tainted, the morally inept or corrupt, indeed, the ‘monsters’ of the cultural imagination’ (Herdt 1993: 17). The reaction of commentators who perceive people with intersex/DSD as threatening prevailing societal values is one of moral panic (Cohen 2002).

Our analysis suggests the importance of personal experience, either in terms of knowing someone with intersex/DSD (reported by only a few commentators) or assumed factual knowledge, on attitudes. The former is consistent with proposals that intergroup contact can reduce prejudice (contact theory), with a meta‐analysis showing the largest effects in respect of contact between heterosexuals and gay men and lesbians (Pettigrew and Tropp 2006). There was also evidence of the martialling of arguments in favour of existing beliefs, as would be predicted by cognitive dissonance theory (Festinger 1957). Comments in respect of Germany are a good example: those in favour of the German law linked it to being a progressive, humanitarian country; those against linked it to negative aspects of Germany's history. Similarly, those referring to religion (specifically Christianity) emphasised either its compassionate aspects or biblical passages relating to the creation of males and females. There was no acknowledgement of the existence of non‐binary alternatives in some other (non‐Western) cultures (Lang and Kuhnle, 2008).

Despite doubts by some editors as to their value (Hermida and Thurman 2008), digital comments have been described as ‘grassroots journalism’, allowing public expression and participation in news‐making to a far greater extent than previously possible through channels such as letters to newspaper editors (Bou‐Franch 2013, Brossoie *et al*. 2012, Diakopoulos and Naaman 2011, Jönsson and Örnebring 2011). Research based on content analysis of such comments is in its infancy, compared with content analysis of the ‘authoritative’ voice of traditional news media (Freeman 2011, Markens 2012) or other aspects of online commenting (Brossoie *et al*. 2012). However, such analysis provides insight into lay understandings and views around the issues involved (Koteyko *et al*. 2013), in this case, sex/gender as binary and overlapping or distinct constructs.

One study categorised motives for online news comment‐writing as being variously information (for example, educating others, answering or asking questions), personal identity (expressing intense emotion or opinion), entertainment (humour, debate) and social interaction (for example, gauging community reactions, persuading others) (Diakopoulos and Naaman, 2011). All were evident in the comments analysed here. A study of online BBC discussions found most posts contained negative emotions and the most prolific posters expressed negative views (Chmiela *et al*. 2011).

Previous analyses of comments to online news stories have described them as frequently provocative, aggressive, negative, impolite, insensitive, racist or sexist, albeit countered by thoughtful commentators who provide a ‘voice of reason’ (Diakopoulos and Naaman, 2011: 136), ask questions, offer different views and challenge socially unacceptable statements (Beyers, 2004, Brossoie *et al*. 2012, Markens, 2012, Neurauter‐Kessels, 2011). Again, our findings are consistent with this and reveal the ‘multiparty’ (Bou‐Franch, 2013) nature of such comments.

The relatively few studies of reader comments have taken different methodological approaches, including quantitative analysis of word frequencies (Koteyko *et al*. 2013), detailed discourse analysis of a relatively small number of comments (Bou‐Franch 2013) and content analysis of themes and patterns in the material (Brossoie *et al*. 2012, Freeman 2011), the approach predominantly taken in our analysis. However, to do this without acknowledging the interactional nature of the data would be to miss something, and we detected individuals with opposing views and different styles, from repeated comments making the same point, to a far more varied range of comments from one person. Future studies might investigate whether focusing on the ‘conversations’ of prolific commentators is an effective way to quickly identify the main themes and commentary styles in material such as this.

As with all such analyses, limitations can be identified, most importantly that commentators are identified by usernames. Apart from those who specifically highlighted some aspect of their identity (for example, particular area of expertise or personal knowledge of an individual with intersex/DSD), we know nothing about them or their location (Neurauter‐Kessels 2011). We do know they were responding to a story in the UK's most used online news source (Ofcom 2013), but, perhaps significantly, relatively few commenting on the story said they had been previously unaware of people with intersex/DSD. In contrast, the few studies of parents of children born with intersex/DSD highlight their absence of knowledge (Gough *et al*. 2008), suggesting the commentators were unusual in knowing about the issue or were unwilling to admit their ignorance to their presumed audience. However, it has been suggested that, despite the fact that commentators are writing for an audience and free to play with identity and deceive: ‘these ‘fabrications’ still tell us something about the manner in which specific social and cultural ideas … are constructed’ (Hookway 2008: 97).

Our starting‐point was an interest in online responses to a challenge to binary ‘sex‐class placement’ (Goffman 1977) in a context of rising societal openness to a range of sexual identities (Roen 2004), growing medical and scientific knowledge of the aetiology and classification of intersex/DSD (Davies *et al*. 2011), and increasing information availability and connectedness between people with intersex/DSD afforded by the Internet (Davis 2015, Kerry 2011). Despite the limitations outlined above, the animated and at times almost visceral comments and debate for and against the German law suggest that social classifications as male or female, even if questioned, remain fundamental in the early 21^st^ century. Davis suggests:

[T]he interactional level of gender structure is where relationships and expectations concerning gender are formed. It's also where individuals reinforce or challenge the gender structure, with assistance or resistance from others.

(2015: 117)

We argue that our analysis of these comments provides a succinct demonstration of these expectations and processes in action.

## Notes

1 Given the problematic nature of the terms ‘sex’ and ‘gender’, our literature review aimed to use the same term as the authors of the original papers.

2 We are also aware that, like others (for example, Paechter, 2003), we are non‐intersex/DSD people writing about people who are intersexed/have DSD, and in doing so have aimed to focus on what the interactions and debates within the BBC comments tell us about society, rather than about intersex/DSD people – see Koyama (2011) and Davis (2015: 189).

3 See, for example BBC (2014) and *The Guardian* (2014).

4 In Germany births must be registered within a week. See Angloinfo Germany (2016).

5 The BBC (2016) website notes the right to fail comments that are abusive or (including using swear words, harassing, threatening or causing distress or inconvenience, trolling, infringing others' rights, attempting to impersonate somebody) or offensive.

6 Those who may appear to be genuine participants but really intend ‘to cause disruption and/or to trigger or exacerbate conflict for the purposes of their own amusement’ (Hardaker 2010).
